# Molecular Imprinting Strategies for Tissue Engineering Applications: A Review

**DOI:** 10.3390/polym13040548

**Published:** 2021-02-12

**Authors:** Amedeo Franco Bonatti, Carmelo De Maria, Giovanni Vozzi

**Affiliations:** Research Center ‘Enrico Piaggio’ and Department of Ingegneria dell’Informazione, University of Pisa, 56122 Pisa, Italy; amedeofranco.bonatti@phd.unipi.it (A.F.B.); carmelo.demaria@unipi.it (C.D.M.)

**Keywords:** tissue engineering, molecularly imprinted polymers, scaffolds

## Abstract

Tissue Engineering (TE) represents a promising solution to fabricate engineered constructs able to restore tissue damage after implantation. In the classic TE approach, biomaterials are used alongside growth factors to create a scaffolding structure that supports cells during the construct maturation. A current challenge in TE is the creation of engineered constructs able to mimic the complex microenvironment found in the natural tissue, so as to promote and guide cell migration, proliferation, and differentiation. In this context, the introduction inside the scaffold of molecularly imprinted polymers (MIPs)—synthetic receptors able to reversibly bind to biomolecules—holds great promise to enhance the scaffold-cell interaction. In this review, we analyze the main strategies that have been used for MIP design and fabrication with a particular focus on biomedical research. Furthermore, to highlight the potential of MIPs for scaffold-based TE, we present recent examples on how MIPs have been used in TE to introduce biophysical cues as well as for drug delivery and sequestering.

## 1. Introduction

The design and fabrication of new materials showing improved biological performance (e.g., cell and tissue compatibility, antibody mimicking) is the challenge that biomaterial scientists are currently facing. In this context, an interesting research trend is the fabrication of synthetic receptors that show an affinity and specificity similar to those found in biological systems. Such receptors, obtained by transferring molecular or structural information from a substance of interest to a synthetic polymer, can enable the fabrication of new, smarter, and customizable biomaterials that are able to recognize and selectively rebind toxins, cytokines, or growth factors (GFs). This approach may pave the way to the study of the physiological interactions as well as to the development of novel approaches in the treatment of several protein-mediated diseases. A further, even more stimulating step forward in this direction could be gained by creating polymeric systems that are able to selectively recognize more complex structures as double-stranded DNAs, viruses, bacteria, or even cells.

In recent years, particular interest has grown around the topic of molecular imprinted polymers (MIPs) [1]. Mainly developed during the 1970s, MIPs are synthetic receptors able to bind to a template molecule with high selectivity. MIPs offer an attractive solution to replicate biological interactions in synthetic materials, since templates such as DNA [2,3], proteins [4], viruses [5], bacteria [6], or cells [7] can be used. When comparing MIPs with natural antibodies, their key advantage is that the binding sites present high specificity for the template but are also stable and capable of withstanding harsher environmental conditions (e.g., temperature, pH) when compared to natural receptors, thanks to the polymeric component [4,8]. Moreover, binding sites in MIPs can be produced by using virtually any target; this makes the technology cheaper and gives it a higher flexibility when compared to natural antibodies, which require the production of a very specific antibody for the given antigen [9]. Finally, MIPs can be more easily prepared and can be stored for longer periods of time (e.g., several months or even years) without any loss in performance [10]. Thanks to these advantages, MIPs have found applications in many different fields, including biosensing [11,12], drug delivery [13,14], and analyte extraction [15]. On the other hand, the technology still presents some drawbacks, including a relatively low imprinting factor (i.e., the ratio of template binding in the imprinted polymer over its binding in the non-imprinted one [9]), as well as a restricted choice of functional monomers that can be used [2]. Moreover, even though MI technology has been successfully applied to small molecule imprinting, there are still limitations regarding its scale-up to larger biomolecules such as proteins [16,17].

An interesting application of MIPs is in the field of tissue engineering (TE) [18,19]. The term “TE” refers to the application of the principles of biology and engineering for developing artificial tissues, using an approach based on three key elements: cells, biomaterial-based matrixes (i.e., scaffolds) and GFs [20]. One of the major objectives of TE is the fabrication of biological substitutes to restore, maintain or improve tissue functionality, with the final goal of transplantation [21]. In the context of TE, MIPs represent a promising solution to introduce cell-stimulating cues inside the scaffold, so that the engineered construct can better replicate the complexity found in the natural tissue, thus enabling a better integration after implantation.

In this review, we aim to give the reader a deep overview of the applications of MIPs in TE. The review is structured as follows: TE is briefly introduced by describing the main elements of a successful scaffolding approach (e.g., the choice of biomaterials, cells and growth factors). Then, the main design considerations regarding MIPs are discussed, with a focus on how to evaluate the MIP quality, as well as critical constraints when choosing the template, functional monomer, solvent, and cross-linker combination. We then move to describing in detail the relevant fabrication technologies that have been applied to MIPs, by reporting the main examples from the literature that are focused on biomedical applications. Finally, we describe the recent applications of MIPs in combination with the TE approach and give a perspective for further development in both fields.

## 2. Tissue Engineering: A Brief Overview

### 2.1. Scaffolds and Biomaterials in TE

The scaffold is the key element in scaffold-based TE. It is a three-dimensional (3D) porous structure that provides mechanical support for growing cells and developing tissue [22,23]. Importantly, a scaffold should present a series of ideal properties, including: (i) biocompatibility (i.e., support cell activities without any local or systematic toxic effects to the host system); (ii) the ability to mimic the host tissue mechanical properties (e.g., Young’s modulus, compressive strength, tensile strength) to optimize load transfer to the tissue; (iii) tailorable surface properties (e.g., roughness and hydrophilicity); (iv) promoting cell activities, including adhesion, migration, proliferation and differentiation (i.e., being bioactive); (v) providing cells with suitable and extra cellular matrix (ECM)-like cues; (vi) being bioresorbable with tunable degradation kinetics; (vii) integrating properly with the host tissue after implantation; and (viii) being easily processable in a wide variety of shapes and sizes [24].

To achieve these target properties, a first important factor to consider is the choice of biomaterials. In general, biomaterials for TE can be classified into two categories—synthetic and natural polymers. Synthetic polymers, including, for example, polycaprolactone (PCL), poly lactic-co-glycolic acid (PLGA), polylactic acid (PLA), present good mechanical properties, making them ideal for load-bearing applications such as those related to the bone tissue. Moreover, they present a good processability, tailored degradation kinetics, and low production costs. On the other hand, synthetic polymers show reduced biological performance [25]. In contrast, natural polymers (e.g., alginate, chitosan, collagen, silk fibroin, hyaluronic acid, decellularized ECM) present good biocompatibility, as well as the ability to direct cellular activities, but they also share high production costs and a reduced processability [26]. For these reasons, research has tried to develop novel bioartificial polymers obtained by combining synthetic and natural polymers in the form of blends, interpenetrating polymer networks (IPNs), and composite polymers to enhance the cell–scaffold interaction and improve their biocompatibility while retaining the performing mechanical properties thanks to the synthetic component [27].

### 2.2. Cell Types and Growth Factors Selection in TE

In the classic paradigm of scaffold-based TE, after the fabrication of the scaffold, cells are seeded onto and into this structure, where they should start producing an ECM-like matrix. Different cell sources have been studied for TE applications, including mature cells taken directly from the patient. Although this solution has been extensively applied, critical issues still remain, including the invasiveness of the cell collection process and the risk of cells being damaged [28]. An alternative solution to these problems is the use of stem cells, which can be expanded in vitro and differentiated into multiple cell lineages to be used to regenerate various types of tissues [29]. Importantly, this choice is strictly dependent on the target tissue. For example, the main cell sources commonly used in bone TE include bone mesenchymal stem cells, adipose-derived stem cells, and muscle-derived stem cells [30,31,32].

In addition, to provide support for cell growth, scaffolds can also be used to deliver GFs or drugs to the sites of repair, in order to speed up the recovery process. For example, some of the most used GFs for bone TE include vascular endothelial growth factor (VEGF), fibroblast growth factors (FGFs), bone morphogenic proteins (BMPs), and β-glycerophosphate. VEGF and FGFs can promote angiogenesis by recruiting endothelial cells and osteoblast, respectively. Moreover, BMPs and β-glycerophosphate are able to guide osteogenesis through osteoprogenitors and mesenchymal stem cell differentiation to osteoblasts [33]. These molecules may be directly incorporated inside the material, but also attached to the scaffold surface. The main challenges to overcome when adding GFs to a scaffold include: (i) controlling the release to match the kinetic of physiological processes, (ii) independently release different GFs at the same time, and (iii) maintaining the molecule activity after processing [34,35].

## 3. Molecular Imprinted Polymers: An Overview

### 3.1. The Basic Principle of Molecular Imprinting

**Figure 1:**
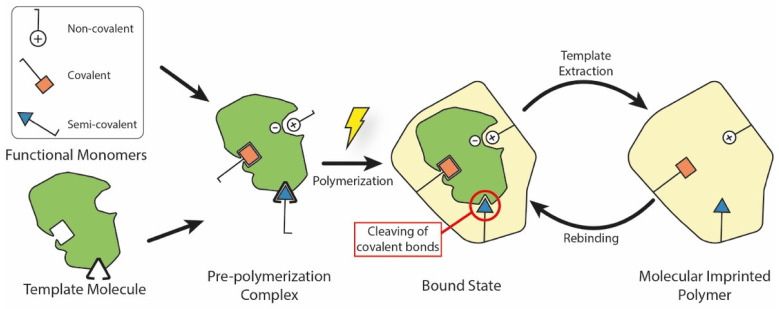
Main elements and fabrication steps to produce a molecularly imprinted polymer. During the pre-polymerization step, different chemical bonds may form between the template molecule and functional monomers. In the semi-covalent case, covalent bonds are cleaved after polymerization, leaving accessible binding sites inside the MIP. During the rebinding step, the template interacts with these sites using non-covalent interactions.

Generally speaking, a MIP is a polymeric system which presents binding cavities to a specific template. Although many different fabrication techniques have been proposed throughout the years, all of them share the same underlying approach which is summarized in Figure 1. Here, the MIP is obtained by using in situ co-polymerization of functional monomers and cross-linkers around templates (or targets). After polymerization, the templates are extracted from the resulting polymeric network (e.g., by washing it using a solvent), leaving permanent cavities of the original template that correspond to its shape, size, and orientation. These represent accessible sites for new targets to bind to, through a mechanism similar to the ‘lock-and-key’ one found in biological systems [4].

Based on the interactions between the functional monomer chemical groups and templates, MIPs have been classified into covalent, non-covalent (e.g., hydrogen bonding, van der Waals or coulomb forces, hydrophobic interactions), or semi-covalent [36]. In covalent MIPs, covalent bonds between the template and functional monomers are formed before polymerization, cleaved during the template removal step, and then reformed during rebinding. In general, this technique yields more defined and more homogeneously distributed cavities, but the required procedure is complex and time-consuming; moreover, since covalent bonds are required, there is a limited monomer–template combination selection available [37]. In the non-covalent approach, the binding sites are formed by the self-assembly of the monomer and template molecules in the pre-polymerization mixture, and are then fixed after polymerization [38]. Non-covalent bonding MIPs are easier to prepare and the templates can be readily removed. However, non-covalent cavities may not be as homogeneous as the covalent ones. Finally, semi-covalent MIPs combine the two previous methods: firstly, covalent bonds are created between the template and the functional monomer; after polymerization, the template is removed by cleaving these bonds, and then the analyte attaches with its binding sites only in a non-covalent fashion [19].

### 3.2. Assessing the MIP Quality

The quality of a synthetic receptor mechanism can be assessed quantitatively based on three main parameters: (i) the binding affinity *BA*, which is a measure of how well a ligand binds to the receptor macromolecule; (ii) the selectivity, which can be defined as the ability to bind specifically to the ligand instead of other competitive molecules; and (iii) the binding capacity *BC*, which represents the ligand bound to the MIP per mass of the imprinted polymer [14]. Mathematically, the binding process can be represented by a reversible reaction (Equation (1)):

(1) [M]+[L]↔[ML]

where *[M]* is the concentration of the MIP binding sites, *[L]* is the concentration of the free template (or ligand), and *[ML]* is that of the bound template (concentrations measured in g/mL). Typically, these quantities are measured by incubating the template and imprinted polymer and gathering data after the binding equilibrium has been reached. Then, the free template concentration *[L]_eq_* can be measured (by using for example UV absorption), while the bound template concentration *[ML]_eq_* can be derived by subtracting the free template concentration from the known initial template concentration *[L]_i_* [39]. Starting from this simple model, the *BA* is usually expressed in terms of the dissociation constant *K_d_* (in g/mL) as in Equation (2):

(2) $$K_{d}=\frac{{\left[M\right]}_{eq}{\left[L\right]}_{eq}}{{\left[ML\right]}_{eq}}$$

A receptor with low values of *K_d_* has a poor *BA*, while higher values represent an enhanced *BA*. The selectivity is measured by adding a competitive molecule to the template-MIP mixture, and is expressed in terms of the a-dimensional separation factor *α* (Equation (3)):

(3) $$\alpha =\frac{K_{d_{i}}}{K_{d_{j}}}$$

where *K_d,i_* and *K_d,j_* are the *K_d_* values for the template and competitive molecule respectively [40]. Finally, the *BC* can be evaluated as the ratio between the template mass bound to the MIP *m_ML_* (in g) and the mass of the polymer *m_M_* (in g). If *V_sol_* is the batch solution volume (in ml), we can write Equation (4) [41]:

(4) $$BC=\frac{m_{ML}}{m_{M}}=\frac{\left({\left[L\right]}_{i}-{\left[L\right]}_{eq}\right)\times V_{sol}}{m_{MIP}}$$

### 3.3. Material Choice Considerations

One of the most important parameters to choose for MIP production is the correct combination of template and functional monomer. Monomer selection depends on the template it is to be imprinted with, since it should present functional groups that are able to bind to the target molecule. For instance, if the template molecule contains basic groups, the monomer should have acidic functional groups, and vice versa. These interactions should be strong both before and after polymerization to yield higher *BAs* [42,43]. Furthermore, it is also important to note that since the interaction between the two is controlled by an equilibrium process, an excess of monomer should be used in order to shift the equilibrium towards the associated state [44].

Besides the monomer–template combination, the crosslinkers and solvent used during the reaction are also both important. During polymerization, the crosslinker is used to fix the functional monomer–template complex, creating a rigid and stable polymer network. The type and amount of crosslinker strongly influences the MIP selectivity and *BA*, as well as the MIP mechanical stability. For example, too low a concentration may result in a mechanically unstable polymer due to a lower cross-linking degree, while too high a cross-linker concentration may reduce the number of recognition sites per unit mass, lowering the MIP *BA* [45]. Regarding the solvent, in general, its choice should not interfere with the monomer–template complex (i.e., there should not be any chemical interactions between the two). However, its type is also dependent on the template that the MIP should be able to bind to. Historically, non-polar solvents, such as chloroform, methanol, and toluene, have been preferred to polar ones, such as water, for small molecular weight imprinting. For these types of templates, water is a ‘bad’ solvent since it will compete for any hydrogen bonding sites on the templates and monomer, affecting the adsorption properties, especially for non-covalent MIPs. On the other hand, proteins are usually unstable and/or insoluble in non-polar solvents; as a result, aqueous solvent is needed [10,16].

Examples of commonly used templates in protein imprinting include bovine serum albumin (BSA), lysozyme, and bovine hemoglobin (BHb). These are often combined with acrylamide (AAm), methacrylic acid (MAA), acrylic acid (AA), and N-isopropylacrylamide (NIPAAm) as functional monomers, while using N,N’ methylenebisacrylamide (N,N’ MBA) or ethylene glycol dimethacrylate (EGDMA) as cross-linkers [10,46].

## 4. Fabrication Strategies for MIPs

### 4.1. Bulk Imprinting

The term “bulk imprinting” (BI) refers to an MIP fabrication strategy in which the template is imprinted directly in the whole three-dimensional synthetic network. The imprinted bulk polymer needs to be post-processed by crushing, grounding, and sieving it to an appropriate size [47,48]. The technique has received attention mainly because of its simplicity and because it represents a cheaper alternative to other imprinting methods that require additional, more complex steps and equipment. However, the main limitation lies in the post-processing, which can be time consuming and yields only a small fraction of the original polymer as usable material [37]. Moreover, the method works best for the detection of small molecules, but is not favorable for larger bioanalytes such as cells and micro-organisms because of slow bulk-diffusion [49].

#### Particle Imprinting

To overcome the limitations of classic BI, different polymerization methods have been proposed to directly create micro- and nano-sized imprinted particles, in what is called particle imprinting (PI) [10]. PI methods include emulsion polymerization, suspension polymerization, precipitation polymerization, and seed polymerization. These methods have already been extensively reviewed elsewhere [19,37,50,51], and their main advantages and disadvantages are briefly summarized in Table 1. The main differences to classic BI are (i) the presence of surfactants/stabilizers, and (ii) the much lower monomer and template concentration in the pre-polymer solution [10].

The use of PI with hydrogel materials is particularly interesting for biomedical applications. Hydrogels are insoluble, crosslinked polymer networks with the ability to absorb significant amounts of water. Thanks to this property, their mechanical properties are close to those of biological soft tissues, which makes them good material candidates for TE applications [58]. In this regard, Pang et al. synthesized PAAm gel beads with a diameter ranging from 150 µm to 280 µm, using inverse suspension polymerization. The beads were imprinted with BSA, and when compared to non-imprinted beads, the imprinted ones showed a much higher *BC* (around 55 × 10^3^ µmol/g versus 10 × 10^3^ µmol/g, respectively). The authors proposed that the imprinting effect was due to both non-covalent bonds (i.e., hydrogen bonds) between the cavities and the template, as well as steric effects [59,60]. On a similar note, Zhang et al. proposed the imprinting of an IPN made of calcium alginate and hydroxyethyl cellulose, in order to increase the mechanical performance of the micro-spheres and reduce their swelling. The beads were then imprinted with BSA using emulsion templating [61]. Interestingly, Herrero et al. developed a method for creating protein-imprinted calcium-alginate microcapsules using ionic gelation, and without the addition of any further chemicals. The biocompatible capsules were imprinted with BSA, and the results showed that they presented higher *BCs* (1.5–3 mg/g) when compared to other imprinting techniques [62].

### 4.2. Surface Imprinting

As an alternative strategy to BI and PI, in surface imprinting (SI) the template binding sites are obtained on the polymer surface. These can be created by synthetizing a thin polymer film using techniques similar to those of BI and PI, or by attaching the template on the substrate surface and then proceeding with the polymerization [10]. When compared to BI, SI recognition sites are more easily accessible, even for larger molecules such as proteins. However, there is also a reduction in the number of imprinted sites, which lowers the sensibility [47].

#### 4.2.1. Soft Lithography

The term “soft lithography” is an umbrella term referring to many different microfabrication technologies, including, but not limited to, microcontact printing, replica molding, and microtransfer molding [63]. The general soft lithographic process can be divided into two main sub-processes: firstly, the fabrication of the elastomeric stamp (from which the name ‘soft’ derives) starting from a so-called ‘master’, which is often obtained using a lithographic technique. To obtain the stamp, a pre-polymer solution (typically poly(dimethylsiloxane), or PDMS) is cast onto the master and polymerized. Finally, the fabricated stamp is used to pattern specific geometries with micro- and nano-scaled features (ranging from 30 nm to 100 µm) [64,65].

**Figure 2:**
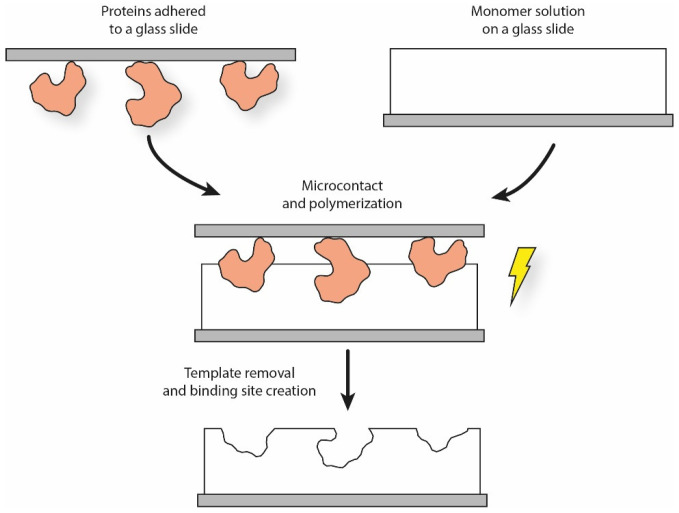
Simplified schematics of the micro-contact printing procedure for proteins.

The soft lithographic procedure can be readily adapted to MIP production. Once the elastomeric stamp is produced, a typical solution is to make the templates adhere to it by self-assembling through weak interactions. The template stamp is then softly pressed over the functional monomer solution for a certain period of time, leading to the creation of patterned surface structures (Figure 2). In the case of cells, cellular suspensions can be expanded onto the stamp surface, thus forming a continuous layer to increase the imprinting density [49].

Interestingly, Ren et al. used a simplified micro-contact printing process to create MIPs for bacteria recognition. The authors spin-coated a PDMS mixture over a glass slide, which was then pre-cured and pressed over a template stamp (i.e., a glass slide with bacteria on it). After curing and eliminating any template residues, a polymeric film was obtained, which showed superficial binding cavities corresponding to the sites previously occupied by the bacteria. The authors also analyzed the effects of both surface treatment protocols (i.e., silanization) and template inactivation (using formaldehyde, ethanol, bleach, ethanol peroxide, and UV). Results showed that the SI procedure facilitated the bacteria capture based on their shape and size, but also thanks to the chemical interaction imparted by the surface treatments [66,67].

Interestingly, SI by soft lithography was also applied to TE. In the work by Vozzi et al., a novel microfabrication technique called Soft-MI was used to produce functionalized TE scaffolds. Briefly, PDMS molds were fabricated using a lithographic technique, and their hydrophobic surfaces were modified to promote gelatin binding. Fibroblast cells were cultured onto the molds, which were then casted with a poly-methyl methacrylate (PMMA) solution to obtain a cell imprinted scaffold for TE applications. The protein- and cell-imprinted scaffolds were able to stimulate cellular processes, including adhesion, proliferation, and differentiation [68].

#### 4.2.2. Template Immobilization

Another approach to SI is template immobilization. This technique differs from soft lithography because the template does not adhere to the stamp through weak interactions, but it is immobilized on it via chemical bonding [4]. The stamp is then exposed to the functional monomer solution, and the polymerization is carried out. Finally, the stamp support is removed using, for example, an appropriate solvent [69]. Template immobilization is particularly suited for proteins since the molecule retains its conformation during the imprinting process. Moreover, the binding sites are formed close to the MIP surface, leading to faster recognition [49].

Shiomi et al. developed a method for template immobilization on silica beads. Specifically, the authors covalently immobilized hemoglobin on the modified beads surface; then, the chosen monomers were polymerized onto the templates, which were finally removed using an acid solution. The hemoglobin-imprinted beads showed excellent selectivity when compared to beads obtained through a free-template protein [70]. Furthermore, using a similar protocol, Bonini et al. developed human serum albumin (HSA)-imprinted polymeric beads. The results showed good HSA recognition, mainly thanks to shape interactions, and the beads’ HSA binding ability both in simple and complex samples such as biological fluids [71]. In a more recent work, Wang et al. covalently bonded a glycoprotein template onto a glass slide whose surface was modified with boronic acid. A hydrophilic coating with a tunable thickness was deposited onto its surface, and the template was then removed using an acid solution containing sodium dodecyl sulfate. Moreover, thanks to the presence of boronic acid, the proposed MIP presented a dual mode for template rebinding: a high affinity mode at acid pH ($K_{d}=2.7\times 10^{-7}$ at pH=3), and a low affinity one at basic pH ($K_{d}=6.6\times 10^{-9}$ at pH=9) [72].

#### 4.2.3. Grafting

A third and final method for SI is surface grafting. Its main difference with template immobilization is that the template units are not bound covalently to the support, but they are adsorbed to its surface thanks to functional groups already grafted on it [49]. For example, Moreira et al. described the imprinting of a polymeric film, poly(o-aminophenol) (PAP), with myoglobin, a cardiac biomarker for ischemia, to create a biosensor. The MIP was obtained by adsorbing the protein onto a gold electrode and using electro polymerization to obtain the PAP. The proteins on the outer layer were then enzymatically removed and washed away, leaving open binding sites. The obtained sensors were tested using electrochemical techniques, and the results highlighted a short measuring time, high accuracy and good selectivity [73]. In another work, Lin et al. created protein-imprinted polymeric films to be used as artificial antibodies. The authors investigated several proteins (including lysozyme, ribonuclease A and myoglobin) to compare the optimal polymer composition for different targets and to create sensor platforms for each protein. The proteins were adsorbed on the surface of glass slides pretreated with hexamethyldisilane, and these stamps were then contacted with different monomer solutions; photopolymerization was finally carried out to obtain the MIPs [74].

### 4.3. Epitope Imprinting

MI for small molecules is well-established; on the other hand, protein imprinting still remains a major challenge [75]. The main limitations reside in the intrinsic properties of the macromolecules themselves; their size impedes the diffusion in the MIP binding sites, while their complexity implies that there is a higher probability of non-predictable interactions with the synthetic material, resulting in a decrease of specificity [76]. Moreover, the protein shape is strongly sensitive to the environment (e.g., pH, temperature), so well-defined and repeatable recognition sites are hard to produce [77]. To solve these limitations, an elegant solution based on the concept of ‘epitope’ (i.e., the part of a macromolecule which is recognized by the immune system, specifically by antibodies, B cells, and T cells), called epitope imprinting (EI), was proposed [78]. Instead of using the whole protein, only a short peptide sequence (molecular weight in the range 500 to 3000 Da) is imprinted in the polymer; when re-binding occurs, the MIP is able to recognize the whole protein starting from the epitope, resulting in more specific interactions and a faster and cheaper synthetization process. Moreover, since the epitope is a small molecule, monomers and cross-linkers commonly used for small-molecule imprinting can also be used for EI [79].

In this context, Corman et al. proposed the use of EI to create MIPs able to bind to immunoglobulin (IgG, one of the subclasses of antibodies). Specifically, the authors imprinted only an aminoacidic residue of the IgG onto poly(hydroxyethyl methacrylate)-based nanoparticles obtained through microemulsion polymerization. The results showed that the imprinted particles could bind to the IgG much better than the non-imprinted ones; moreover, the selectivity to the protein was increased when exposing the imprinted and non-imprinted nanoparticles to albumin and hemoglobin as competitor molecules [80].

**Figure 3:**
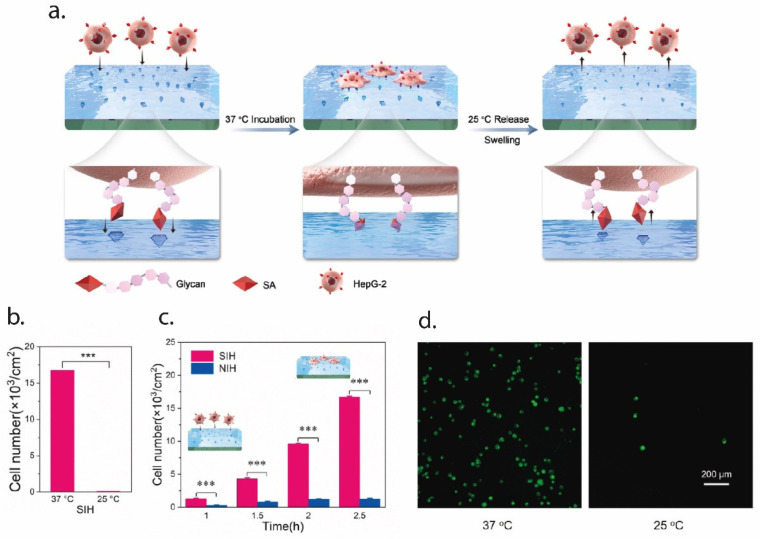
An example of EI for the selective capture and release of cancer cells. In (**a**), a schematic representation of the process. The SA epitope is imprinted over the surface of the PNIPAAm hydrogel; at 37 °C, the epitope is exposed on the surface, so that cancer cells can bind to it. When lowering the temperature to 25 °C, the conformational changes in the thermo-responsive hydrogel cause the cell release, since the SA group is no longer exposed. In (**b**), the efficiency of the cell capture-and-release method, expressed in terms of the cell number, while in (**c**) the capture profile over time compared to the non-imprinted hydrogel (NIH). Finally, in (**d**) the staining of the cell on the hydrogel surface at 37 °C (captured cells) and 25 °C (released cells). Figure modified with permission from [83].

In an interesting paper, Zhang et al. employed EI for the tracking of tumor cells, using EI with a peptide found in the extra-cellular portion of the p32 membrane protein, which is known to be overexpressed in tumor cells. AAm and N,N’ MBA were used as functional monomers and the molecularly imprinted nanoparticles were synthesized using microemulsion polymerization. Results from both in vitro and in vivo studies showed that the MI particles presented a stable and specific targeting of the tumor cells [81]. In a more recent paper, EI was used to create an artificial tumor-specific antigen by using HER2 as a template. HER2 is a receptor for human epithelial growth factor and is over expressed in ovarian cancer cells. The authors fabricated HER2 epitope-imprinted silica nanoparticles for the targeted delivery of the drug doxorubicin (DOX). The novel drug-delivery system was tested in an in vivo mouse model, and the results highlighted the ability of the imprinted particles for targeted delivery of DOX to the cancer cells [82]. Finally, Ma et al. imprinted the surface of a thermo-responsive hydrogel with sialic acid (SA) to selectively capture and release cancer cells, as can be seen in Figure 3. SA is typically overexpressed in the membrane of cancer cells. The thermo-responsive hydrogel (NIPAAm) allowed to tune the capture and release of cells; at 37 °C, the binding sites are all exposed and cancer cells can bind to them, while at temperatures lower than 25 °C the sites change in shape and functionality, so that the cells may be quickly released. Results showed the selective capture of cancer cells (HepG-2) and release by changing the temperature [83].

## 5. Molecular Imprinting for TE Applications

In natural systems, molecular recognition is key at different scale levels [43]. For example, the immune system is able to recognize foreign materials inside the body (using antigens) by using globular proteins (antibodies) that express specific antigen binding sites. At the cell scale, the ECM is composed of fibrillar and amorphous components, which interact with cells via cell surface receptors (i.e., integrins). Cell–ECM interactions can determine cell differentiation and cell growth, cell orientation, secretion of molecules and ECM remodeling [84,85]. Moreover, GFs are bound to the ECM insoluble components, which dynamically regulate their concentration and activity. In this regard, scaffolds have to provide not only the adequate mechanical and structural support, but also actively guide and control cell attachment, migration, proliferation, and differentiation. To do so, the scaffold functionalities should be extended to provide biological signals, including cues to interact with cells as well as the ability to deliver GFs in a controlled way [86]. The following sections describe the literature examples of how MIPs have been used in sTE applications (Table 2).

### 5.1. Molecular Imprinting to Introduce Chemical Cues inside the Scaffold

A key challenge in TE is to guide cell behavior and tissue formation by fabricating functionalized scaffolds that are able to promote cell-biomaterial interactions. Such interactions require material biomolecular recognition by cells, which can be imparted by introducing chemical cues inside the scaffold. In this context, MI has already been employed to introduce carriers for these biomolecules.

Starting from a previous work [68], Criscenti et al. combined Soft-MI with electrospinning to create bioactive scaffolds for TE. Specifically, the surface of electrospun mats was imprinted with different proteins and GFs (e.g., FGF-2, BMP-2), and then cultured with cells. The results showed that the imprinting technique strongly influenced cell behavior in terms of cell proliferation, cell number, and metabolic activity [91]. In another study, Rosellini et al. proposed the use of EI in combination with scaffolding for TE applications. The authors used precipitation polymerization to obtain MAA-based microgel beads, imprinted with a peptide sequence exposed by fibronectin. The beads were then used to functionalize films by simple deposition, to create a novel bioactive scaffold. The authors showed that the MI beads could selectively bind to both the single peptide sequence and the whole fibronectin molecule, and, when used for scaffold functionalization, could promote cell adhesion and proliferation [92]. Pan et al. used imprinting of a PNIPAAm surface to create thermo-responsive cell culture substrates for cell sheets TE [93]. The hydrogel was imprinted using a cell-adhesive peptide, RGDS (a tetrapeptide with the sequence Arg-Gly-Asp-Ser), which is a commonly used peptide to promote cell adhesion. By rebinding the surface with the peptide, a single cell layer could be cultured at 37 °C. By lowering the temperature, the cultured cells could detach spontaneously as the surface of the substrate changed from hydrophobic to hydrophilic. The intact cell layer was then harvested non-invasively together with its ECM. In a more recent paper by the same authors, microcontact printing was used to imprint a surface with RGD. The authors showed the ability of the imprinted surface to reversibly bind and re-bind the peptide, effectively creating a platform with tunable cell-adhesion properties [94].

### 5.2. Molecular Imprinting to Control Cellular Activities through Physical Cues

**Figure 4:**
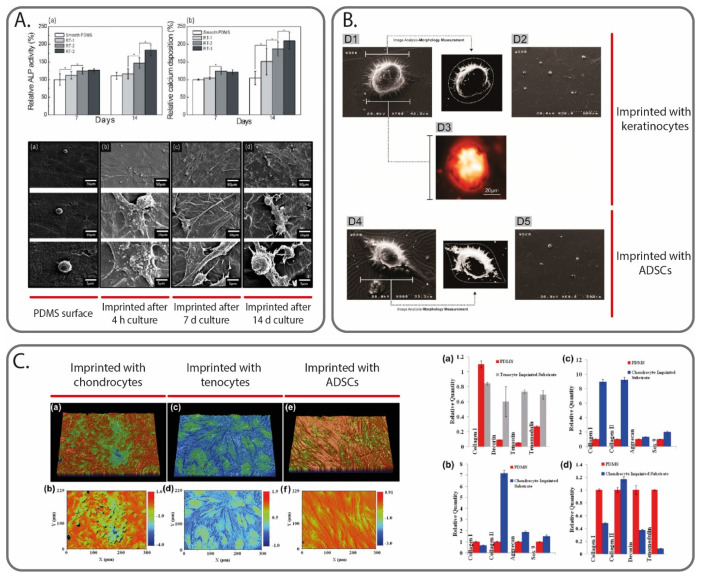
Cell MI to create physical cues able to guide cell activities. In (**A**), ALP activity and calcium deposition measured at 7 and 14 days for osteoblast-like cells culture on smooth PDMS surface, and on PDMS surface imprinted with the same cell type after different culturing times (4 h, 7 days and 14 days). Image reprinted with permission from [90]. In (**B**), atomic force microscope images of ADSCs cells cultured on a keratinocytes-imprinted and ADSCs-imprinted silicone substrate. Image reprinted with permission from [100]. In (**C**), profilometry images of the PDMS substrates imprinted with different cell types (chondrocytes, tenocytes and ADSCs), alongside the gene expression results of different cell cultures on the substrates (specifically, (**a**,**b**) ADSCs, (**c**) fibroblasts and (**d**) tenocytes). Image reprinted with permission from [101].

Another solution to guide cellular activity includes the introduction of physical cues inside the scaffold that can replicate the natural ECM environment. Interestingly, MIPs have already been extensively studied as a tool to influence cells by using them as templates. Among the different MIP fabrication strategies, micro-contact imprinting using thole cells represents the most used method [95,96,97]. For example, DePorter et al. reported a simple and inexpensive procedure, in which mammalian cells were cultured onto a glass slide, and then AAm was poured onto it, leaving cavities. The imprinted cell features on the hydrogel surface were found to act like cues to promote cell adhesion and growth [98]. In another work, Mahmoudi et al. reported the use of MIPs to direct the differentiation of stem cells. The authors cultured both spherically (matured) and spindle-shaped (dedifferentiated) chondrocyte cells. A PDMS solution was poured on top of the chondrocyte cells and was removed at the end of the curing step. The PDMS surface was seeded with adipose-derived mesenchymal stem cells, which changed to the shape of the cavities; more importantly, it also presented the molecular characteristics (i.e., gene expression) of the template cell types [99]. In a later article, Mashinchian et al. presented an in vitro study in which stem cells cultured onto a surface imprinted with mature keratinocyte adopted keratinocyte-like morphologies and expressed keratinocyte marker genes and proteins (Figure 4B) [100]. On a similar note, Bonakdar et al. also studied the effects of cell-imprinted substrates on stem cells (adipose-derived mesenchymal stem cells). Specifically, the cell shape analysis, as well as the upregulation/downregulation of specific genes, confirmed the effects to induce shape change and differentiation (Figure 4C) [101]. Finally, Jeon et al. fabricated a patterned surface model using a casting approach, by replicating the shape and patterns of proliferated cells, which were acquired from osteoblast-like cells cultured for different periods of time (4 h, 7 and 14 days). Using the negative surfaces, the same cell type was cultured on them, and various biological activities were monitored (cell viability, alkaline phosphatase activity, calcium deposition). The results showed a significant enhancement of all these activities (Figure 4A) [90].

### 5.3. Molecular Imprinting for Drug Delivery and Sequestering

The ability of MIPs to bind a template molecule with high selectivity makes them ideal candidates for drug delivery systems (DDS) [102]. DDS are able to deliver a specific drug to its target (i.e., local delivery), without damaging non-target sites [103]. Moreover, they can be employed to release drugs over an extended period of time without delivering too low or too high a dose (controlled release), in order to achieve the maximum therapeutic effect [104]. As an example of the application of MIPs to DDS, Mao et al. have developed MIP nanospheres by precipitation polymerization, which are able to bind the antibacterial vancomycin and release it over an extended period of time (over 18 days) and with higher release rates at lower pH values. The nanospheres are a promising approach to developing DDS to prevent bacterial infections [105]. In another recent example, Gu et al. employed MIPs in drug delivery for cancer treatment. The authors reported the use of a MIP-based prodrug delivery system, which were able to accumulate and selectively bind to the tumor site, and locally release the prodrug in an in vivo tumor mouse model. Results from fluorescence imaging showed a high targeting capacity and long retention times when compared to the non-imprinted control. Moreover, the tumor growth rate was significantly reduced with the proposed method [106].

In the context of TE, DDS can then be employed not only to deliver GFs to the forming tissue, but also other drugs to enhance the tissue regeneration process. For example, in [107] the authors reported the use of an antibiotic (vancomycin) used as a template for wound healing applications. After production, the MIP was encapsulated in an alginate matrix to obtain an antibacterial wound dressing. In vitro drug release and biological activity studies using Gram-positive bacteria showed that (i) the antibiotic release was significantly slowed down after encapsulation in the alginate matrix, and (ii) the amount of drug released in the first 24 h was sufficient to inhibit the bacteria. As a result, the MIP-based DDS can guarantee sustained and effective antibiotic release without changing the dressing. In a recent paper, Koudehi et al. proposed the use of MIPs to produce a DDS for skin wound healing. Specifically, they produced MIP nanoparticles using gentamicin as a template, which were then electrospun alongside PVA and gelatin. The electrospun fiber mat was then used for both in vitro cytotoxicity and drug release tests, as well as in vivo implantation in a rat model. After 14 days, the novel composite biomaterials showed accelerated wound healing when compared to electrospun PVA/gelatin fibers and an untreated control [87].

Finally, an equally important application of MIPs to TE scaffolding is the sequestering of biomolecules to promote tissue regeneration. GFs are naturally over-released near the site of injury, and as a result scaffolds can leverage this process by sequestering these biomolecules to create a proper microenvironment for regeneration [108,109,110]. In this context, MIPs represent a promising solution to this challenge, thanks to their specificity. For example, Teixeira et al. used EI to produce imprinted nanoparticles able to sequester endogenous GFs (TGF-β3)—polyacrylamide beads with inverse microemulsion polymerization. The results showed that the nanoparticles could selectively bind the GF both in noncompetitive assays and in complex human fluid (platelet lysate). Human adipose stem cells, when incubated with platelet lysate and the imprinted nanoparticles, resulted in a higher collagen matrix deposition when compared to the control (non-imprinted nanoparticles) [111].

Besides GFs, other molecules may be secreted in the site of injury which can impede the regeneration process. MIPs able to scavenge them from the environment can help increase the speed of the regeneration process. In this context, Cristallini et al. used the MI approach to create particles of MAA and poly(ethylene glycol) ethyl ether methacrylate, imprinted with matrix metallopeptidase 9 (MMP-9). This enzyme is involved in the early adverse phases of stroke in the myocardium, and its removal may promote better tissue regeneration. The nanoparticles showed a good ability to entrap the template MMP-9. Moreover, after spraying the MIPs onto a scaffold, the whole biofabricated structure, which presented features at the nano, micro and millimeter scales, showed a good ability to replicate a cardiac ECM-like environment [88].

## 6. Discussion and Future Perspectives

TE represents a novel and promising approach to tissue regeneration, which leverages advanced know-how in different fields, including engineering, biology, biomaterial science and medicine. Using a combination of three main elements, biomaterials, cells, and GFs, the scaffolding approach in TE aims to create living tissues that are able to integrate with the site of implantation and promote tissue regeneration. In order to achieve a successful integration, it is important that the engineered construct is able to replicate the complexity of the natural ECM environment and control cellular activities. In this regard, MI promises to improve the bio-mimicking properties of the scaffold by introducing synthetic receptors that work in a similar way to those found in biological systems. As was discussed in the previous sections, MI has already been applied successfully to TE by introducing chemical and physical cues to the scaffold. It has also been applied to deliver GFs/drugs and sequester endogenous biomolecules to enhance the regeneration process. Moreover, MI applications are not limited only to scaffolds for tissue regeneration and repair, but represent a promising solution for in vitro models, which are able to replicate in vitro the complex tissue environment to better study its physiology and pathology, as well as efficiently test new drug formulations [112].

Besides the highlighted example applications, that are still several open challenges in TE that MI can help to solve.

A major problem when creating a scaffold for implantation is to have enough active cells to seed it. The solution of harvesting adult cells directly from the patient, although straightforward in theory, is not commonly applied in practice since the harvesting process can be invasive and damaging for other tissue. On the other hand, the use of stem cells suffers from the problems of their correct differentiation, as well as keeping their phenotype over time. In this context, the use of MI substrates, although with less differentiation potential when compared to GFs, represents an inexpensive and simple way to expand and differentiate stem cells for TE purposes [113]. In a recent example, Kashani et al. proposed the combination of MIPs and microfluidics devices by using a two-step approach: create a chondrocytes-imprinted substrate using a first microfluidic device, and then culture stem cells over it using a second device. After 14 days in culture, the cells were able to differentiate into chondrocytes, showing a typical spherical morphology. By themselves, microfluidic devices can provide temporal and spatial control over the microenvironment and are able to stimulate multiple cells in parallel with a high-throughput. The addition of MI improves the efficiency by providing strict control over cell placement [114].

The real-time visualization of the progress of the developing tissue is another important aspect in TE. This can give useful insights on the regeneration progress and scaffold degradation after implantation, as well as helping to understand the physiology or pathology of the target tissue in an in vitro model. In this regard, MI has already been applied for fluorescent labeling, and may represent a non-invasive solution to this challenge. Fluorescent labelling of tumor cells has been extensively studied throughout the recent literature, since cancer cells over-express specific membrane molecules which can help identify them from the other cells [115,116,117]. For example, Demir et al. employed carbon nanodots (CDs) in combination with an MIP shell able to selectively bind to glucuronic acid. This is an epitope of hyaluronan, an ECM polymer whose concentration is higher for specific cancer cells (e.g., colon, breast, prostate, lung, bladder, cervix). The CD core produces an intense fluorescence when exited with UV light, and thanks to the selectivity of the MIP shell, it is able to label cancer cells [118]. In another example, Panagiotopoulou et al. produced fluorescently-labeled MI nanoparticles to be used for imaging of both fixed and living human keratinocytes, in order to localize SA and hyaluronan, both indicators of cell malignancy. The nanoparticles showed high selectivity towards their templates, while being non-cytotoxic. Thanks to the ability to produce them at different sizes, the particles were able to bind to both extracellular membrane portions (particle dimensions of 400 nm) as well as inside the cells (particle dimensions of 150 nm) [119]. Besides cell-imaging, MIPs have also been used for direct therapy, by selectively binding and killing specific cell types. In this regard, MI nanoparticles have been recently used for detection and clearing of senescent cells, by leveraging EI for an extracellular senescent cell marker. The MIPs were tested both in vitro and in vivo, and the results highlighted no toxic response as well as the ability to specifically bind to senescent cells. Moreover, the authors also showed a proof-of-concept in vitro application to detect and kill senescent cells by loading the MIPs with specific drugs [120]. Using another approach, Yin et al. used SA as a template for cancer cell detection, by imprinting it onto gold nanorods. The nanorods were able to selectively bind to cancer cells in an in vivo tumor-bearing mouse model. When injected in the blood stream, the nanorods tend to accumulate towards the tumor, which could then be located by imaging. A near-infrared laser was then aimed at the tumor site, and the nanorods absorbed the photon energy and converted it to heat efficiently, thermally killing only the tumor cells [121].

Finally, the binding selectivity of MIPs may be exploited to create biosensors for quick detection of biomolecules related to pathologies. These sensors may be directly embedded inside the scaffold or used in an in vitro model, further increasing the amount of control over the cell microenvironment. For example, a novel EI technique based on electro-polymerization of peptides onto a gold surface was recently used to create protein sensors for the detection of cancer biomarkers [122]. Furthermore, Lakshmi et al. developed an MIP-based electrochemical sensor to detect trimethylamine N-oxide (TMAO). TMAO is a microbiota metabolite which is thought to be associated with several pathologies, including cardiac and kidney chronic diseases. The authors used exploited non-covalent bonding between the template TMAO and the electroactive polymer polypyrrol. The biosensor showed a broad linear detection range, excellent sensitivity, and high selectivity with a fast response time (around 20 min) [123].

Although preliminary examples have been successful, there are several unmet challenges that limit the translation of MI to TE, which need to be addressed in the near future. As was already mentioned, the imprinting of complex biomolecules such as proteins is challenging due to the intrinsic nature of these templates. Moreover, a wider range of non-cytotoxic materials and cell-friendly processing conditions should be investigated, alongside an optimization of the fabrication techniques to yield more consistent results and facilitate the translation to industry.

## 7. Conclusions

MI offers an advanced solution to produce bioactive scaffolds for TE applications, that are able to enhance tissue regeneration. In this review, we provided an analysis of the basis for scaffold-based TE and of the rational design for MIP production. We then moved to describe in detail the different fabrication technologies that have been applied to produce MIP, with a particular focus on literature examples that used biological templates (e.g., proteins, cells, bacteria). We analyzed the main examples of MI for TE scaffolds by showing how MI was used to introduce biological and physical cues inside the scaffold, as well as for controlled and localized delivery of GFs and drugs, and biomolecule sequestering. These examples highlight the flexibility of the MI technique and show how it can apply to TE to better mimic the complex ECM environment.

## Figures and Tables

**Table 1:** Main particle imprinting mechanisms, alongside their advantages, disadvantages and relevant examples from literature.

PI Mechanism	General Approach	Advantages	Disadvantages	Example Ref
Emulsion	The monomer phase is suspended in an immiscible phase (e.g., water). The polymerization takes place inside monomer droplets, which are enclosed in micelles and stabilized by surfactants.	Able to produce nanoparticles. Two possible approaches are available: mini-emulsion (30–500 nm imprinted particle diameter) and micro-emulsion (5–30 nm imprinted particle diameter).	Surfactants and water are both required for the procedure, which can interfere with the template–monomer interactions and lower the selectivity.	[52,53]
Precipitation	Similar procedure to bulk imprinting, but with a higher amount of solvent used. The polymeric chains do not overlap to create a network, but continue to grow individually, until they reach a critical mass and precipitate.	Fast and cheap syntheses of spherical imprinted particles. Easy procedure. High yield and uniform diameter.	A high amount of template is needed.	[54,55]
Suspension	Droplets of the pre-polymerization mixture are suspended in an immiscible phase (e.g., water) in the presence of surfactants. Polymerization takes place inside the droplets.	Spherical imprinted particles. Large-scale production. Highly reproducible results.	Surfactants and water are both required for the procedure, which can interfere with the template–monomer interactions and lower the selectivity. The produced particles are polydispersed in size (a few to a few hundred microns).	[40,56]
Seed	A porous particle (seed) is used as a scaffold structure upon which the polymerization is carried out	Yields monodispersed imprinted particles with uniform diameter	Complex procedure Needs aqueous solvent which may lower the selectivity for non-covalent MIPs	[57]

**Table 2:** Examples of the translation of MI to TE, alongside the clinical application of the specific case.

Brief Description	Fabrication Strategy	Clinical Application	Example Ref
Nanoparticles that can bind an antibacterial drug to release it with control over time.	Precipitation Polymerization	Antibacterial wound dressing	[87]
Nanoparticles imprinted with matrix metallopeptidase 9 (MMP-9), whose scavenging may promote better cardiac tissue regeneration.	Precipitation Polymerization	Cardiac tissue regeneration	[88]
Imprinted particles selective for laminin and fibronectin, used for the functionalization of biomimetic sponges.	Precipitation Polymerization	Cardiac tissue regeneration	[89]
Imprinted PDMS surface with osteoblast-like cells. The surface was seeded with stem cells, which differentiated to towards the imprinted phenotype.	Micro-contact printing	Bone tissue regeneration	[90]
